# Who Actually Dies from Indoor Air Pollution? A Forensic Perspective

**DOI:** 10.3390/diagnostics16071038

**Published:** 2026-03-30

**Authors:** Nicola Pigaiani, Andrea Costantino, Fabio Vaiano, Maria Grazia Fornasari, Ilenia Bianchi, Edoardo Orlandi, Fabrizio Carta, Francesco Ausania, Simone Grassi

**Affiliations:** 1 Unit of Forensic Medicine, Department of Diagnostics and Public Health, University of Verona, P.le L.A. Scuro 10, 37134 Verona, Italy; nicola.pigaiani@univr.it (N.P.);; 2 Forensic Medical Sciences, Department of Health Science, University of Florence, 50121 Florence, Italy; 3 FT-LAB Forensic Toxicology Laboratory, Department of Health Science, University of Florence, 50121 Florence, Italy; 4 Sezione di Scienze Farmaceutiche E Nutraceutiche, Dipartimento Neurofarba, Università Degli Studi di Firenze, Via Ugo Schiff 6, Sesto Fiorentino, 50019 Florence, Italy; 5 Laboratory on the Management of Healthcare Incidents (LOGOS), Department of Health Sciences, University of Florence, 50134 Florence, Italy

**Keywords:** indoor pollution, air pollution, autopsy, post-mortem diagnosis, toxicity

## Abstract

In high-income countries, humans are continuously exposed to indoor and outdoor air pollution. Chronic exposure to these airborne solids and gases from natural or artificial sources is related to higher mortality. The objective of this work is to critically assess whether the association between indoor air pollution and death can support robust causal inference from a strict medico-legal perspective. We conducted a narrative review of existing literature on reported health consequences, autopsy and histopathological findings potentially linked to indoor air pollution exposure, and dose–response relationships and examined their role in criminal liability in Western countries. Despite prevention measures and regulations, establishing criminal liability for indoor air pollution remains arduous beyond a reasonable doubt given associative epidemiological evidence, translational biases, and non-specific autopsy findings. Further research on non-linear models and targeted forensic investigations is warranted.

## 1. Introduction

In high-income countries, poor indoor air quality is a public health threat since 90% of time is spent indoors and the consequence of improved building insulation is poor ventilation and increased dampness [1,2]. Not only can households be polluted, but common workplaces such as offices and shopping malls have been associated with high concentrations of particulate matter smaller than 2.5 µm (PM_2.5_) of 10–44 and 74–164 μg/m^3,^ respectively, and with significant levels of other pollutants (and suspected carcinogenic chemicals), such as toluene and xylene [1]. Indoor pollution is mainly due to human activities, in particular, to combustion (such as tobacco smoking) [3]. Indeed, smoking and e-vaping can release toluene, NO, CO, acrylamide, acetaldehyde, formaldehyde, and particulate matter (e-vaping can also emit metals such as nickel, silver, and copper) [4]. Even cleaning is a source of indoor pollution, since it helps to resuspend settled particles [2]. Moreover, humans themselves are sources of indoor pollution: besides CO_2_, many volatile organic compounds (potentially or known to be carcinogenic—such as benzene) are normally exhaled by healthy individuals [5]. Other common indoor pollutants are those that infiltrate from outdoors (e.g., O_3_), molds/allergens, and cleaning products that can release terpenes, phthalate esters, and polyfluorinated compounds [1,2,3]. Particulate matter is represented by carbonaceous particles, with primary constituents including heavy metals (some of which—such as arsenic, chromium, cadmium and lead—are potentially carcinogenic), endotoxins, polycyclic aromatic hydrocarbons, sulfates, and nitrates [6]. Air can also be polluted by radioactive substances, such as radon: this gas can infiltrate buildings from the surrounding soil/rock (posing a contamination risk, especially to lower floors) or be directly emitted from some building materials (such as concrete, bed stones, and brick) [7]. Furthermore, building materials can also contain other pollutants—for instance, formaldehyde (from formaldehyde-urea foams, adhesives, plywood, and particle board) or fibers, such as asbestos (that, albeit prohibited, is still almost ubiquitous) [1]. The medico-legal relevance of this issue is supported by the many criminal and civil proceedings concerning severe diseases or deaths allegedly caused by polluted closed environments. For instance, about 125.000 annual deaths in Europe are thought to be caused by PM_2.5_, and in the US, each 1 μg/m^−3^ increase in PM_2.5_ concentration is associated with more than 30,000 deaths [5,8]. Forensic experts are called to verify if a proven or alleged exposure to one or more pollutants is a plausible cause of the death or the health disorder that occurred, and this task is particularly complex, since it requires combining pathological data, scientific evidence, and causal reasoning. Therefore, in this paper we aim to review the cornerstones of forensic investigation in these cases and to discuss the main pitfalls.

This narrative medico-legal review focuses on the causal attribution of deaths to indoor air pollution, rather than offering a comprehensive descriptive review or technical guideline, and includes a concise overview of exposure assessment methods and relevant air quality standards.

## 2. Materials and Methods

We conducted a narrative review of the peer-reviewed literature to identify key evidence on indoor air pollutants, their toxicity profiles, their reported health consequences, and autopsy/histopathological findings potentially attributable to indoor air pollution exposure. Studies were selected based on their relevance to medico-legal and toxicology contexts. Electronic searches were performed in PubMed, Scopus, and Web of Science for articles published up to January 2026, using combinations of keywords related to indoor air pollution, household air pollution, particulate matter, volatile organic compounds, combustion products, indoor environmental quality, autopsy, histopathology, sudden death, cause of death, manner of death, toxicology, and forensic medicine. Additional references were identified by screening the reference lists of relevant reviews and key primary studies. Given the aim of integrating mechanistic, pathological, and medico-legal perspectives rather than estimating pooled effect sizes, we adopted a narrative review approach. Studies were excluded if they focused exclusively on outdoor air pollution without clear relevance to indoor environments; lacked any pathophysiological, forensic, or legal-causal dimension; or were conference abstracts, non–peer-reviewed reports, or opinion pieces without primary or clearly described secondary data. Article selection and interpretation were performed independently by at least two authors; disagreements about inclusion or relevance were resolved through discussion and, when necessary, consultation with a third senior author. Because this was not a systematic review, we did not apply formal risk-of-bias tools or conduct quantitative meta-analysis. Instead, we qualitatively assessed study quality and relevance based on clarity of exposure assessment, appropriateness of outcome measures (including autopsy and histology where available), control of major confounders, and explicitness of the causal framework. Particular emphasis was placed on how each study could inform the forensic questions of interest regarding dose–response relationships, mechanistic plausibility, the specificity of pathological findings, and their potential use in individual-level causal attribution.

## 3. Results

### 3.1. Toxicity of Indoor Pollutants

Air pollutants may be inhaled, ingested, or absorbed, and their toxicity can be primary (produced directly by the substance, even at sites distant from the exposure site, as in gastrointestinal symptoms caused by inhaled lead) or secondary (produced by the formation and accumulation of byproducts, as in the case of arsenic reduction) [9]. Since most of them are inhaled, pollutants mainly endanger respiratory health, causing a pro-inflammatory state with neutrophilic inflammation, impaired microbial clearance, surfactant deactivation, a damaged lung endothelial barrier, and increased oxidative stress [2,10]. As previously stated, pollutants are normally solids or gases. Gas toxicity also depends on basic chemical properties such as solubility: if a pollutant is highly soluble in water and an irritant (e.g., SO_2_), it usually causes the most damage to the skin and upper airways; if it is highly soluble but non-irritating, it is able to reach the blood and bind hemoglobin (e.g., CO or NO); while if a gas is less soluble (e.g., O_3_ or NO_2_), it can infiltrate the lower respiratory tract [11]. Regarding solid particles, their toxicity largely depends on their dimensions: for instance, PM_2.5_ can reach the terminal bronchi and the alveoli, from where they can be transferred into the bloodstream, while PM_0.1_ are even more dangerous because they can easily cross the alveolar–capillary barrier, potentially bringing with them elements such as arsenic or polycyclic aromatic hydrocarbons, which are bonded on their surfaces [11]. Regarding fibers, the toxicity (and carcinogenic potential) of mineral fibers depends on their dimensions: long and thin fibers tend to accumulate and be biopersistent so that macrophages fail to clear them, an inflammatory response is provoked, and free radicals are released [12].

### 3.2. Exposure Assessment and Indoor Air Quality Metrics in Forensic Context

Within a forensic framework, exposure assessment integrates environmental data, post-mortem toxicological analyses, and indirect reconstructive evidence with methods selected based on diagnostic suspicion and the temporal window of exposure (acute vs. chronic).

The main indoor monitoring methods include stationary sensors for PM_2.5_/PM_10_ (laser scattering) [13], CO/NO_2_ (electrochemical) [14], VOCs (PID/MOS) [15], formaldehyde [16], and radon (specific detectors such as track-etch detectors) [17], typically integrated with temperature, relative humidity, and CO_2_ as ventilation proxies. A critical distinction exists between environmental monitoring (real-time/integrated air sampling) and biomonitoring (e.g., blood COHb for acute CO, heavy metals in blood/urine, VOC metabolites), the latter providing direct evidence of systemic uptake but often lacking indoor-source specificity [18].

International guidelines, primarily developed for outdoor exposure, are often applied by analogy to indoor settings. The WHO Air Quality Guidelines (2021) [19] recommend PM_2.5_ annual mean < 5 µg/m^3^, 24 h < 15 µg/m^3^; NO_2_ annual < 10 µg/m^3^; CO 24 h < 4 mg/m^3^. The Directive (EU) 2024/2881 [20] mandates PM_2.5_ annual < 10 µg/m^3^ and NO_2_ < 20 µg/m^3^, with monitoring required in public/sensitive buildings, though specific indoor limits remain limited. Extrapolation from outdoor to indoor data is problematic due to differing mixture compositions (higher indoor VOC/combustion dominance), heterogeneous microenvironments (e.g., cooking peaks), and ventilation-driven non-linear exposures. In retrospective cases, dose reconstruction relies on indirect data such as building characteristics (insulation, natural/mechanical ventilation), fuel/appliance types (wood/gas stoves, candles) and household habits (passive smoking, unvented cooking), yielding probabilistic estimates rather than definitive proof.

However, in a forensic context, the approach varies based on the nature of the exposure. For acute or recent exposures (<6 months), environmental monitoring remains a primary diagnostic tool: portable sensors for PM_2.5_, CO, and VOCs over a 24–72 h period can document potentially hazardous concentrations. Conversely, for chronic exposures (>1 year), environmental inspection has limited evidential value, as the current site rarely mirrors the pathogenic one, making historical reconstruction through documentation and witness statements a priority [21].

Regarding post-mortem toxicological analyses, a targeted sampling strategy must be tailored to the specific diagnostic hypotheses. For carboxyhemoglobin (COHb) determination, for example, cardiac blood is preferably analyzed using gas chromatography or UV-Vis spectrophotometry/co-oximetry [22]. Assessment of heavy metal concentrations (Pb, Cd, As, Hg) requires a stratified approach: Inductively Coupled Plasma Mass Spectrometry on hair and nails is utilized to evaluate cumulative exposure, while blood and urine analyses address acute/recent intake. Furthermore, the analysis of solid matrices, such as the kidney and liver, is essential to document systemic bioaccumulation [22]. The measurement of urinary VOC metabolites (e.g., mandelic acid, phenylglyoxylic acid) via Liquid Chromatography–Mass Spectrometry or Gas Chromatography–Mass Spectrometry allows investigation of exposures primarily within the 24–48 h preceding death, despite limitations imposed by post-mortem degradation [23]. Additionally, the detection of black carbon in tissues (placenta, lung, liver) via Transmission Electron Microscopy with Energy Dispersive X-ray Spectroscopy serves as an indirect marker of chronic particulate deposition, though it lacks a robust quantitative correlation with absorbed dose. However, the quantitative correlation between tissue deposition and chronic inhaled dose remains to be validated in autopsy contexts [24].

Evidential constraints remain a central challenge in forensic investigations. The extrapolation of outdoor data to indoor environments is problematic due to compositional differences, with greater indoor dominance of VOCs and combustion sources, heterogeneous microenvironments, and non-linear exposures driven by ventilation. In retrospective cases, dose reconstruction relies heavily on indirect data, yielding only probabilistic estimates. Therefore, standardized investigation of enclosed environments must incorporate: ventilation assessments (e.g., operable windows, extractor functionality), source identification (e.g., fuel/appliance types, maintenance records), air monitoring where feasible (e.g., existing sensors, portable PM_2.5_/CO/radon detectors), and structured anamnesis (e.g., occupant habits, symptom progression via medical records/witnesses).

### 3.3. Reported Health Consequences

Since most indoor pollutants are inhaled, respiratory disorders represent most of the health consequences: for instance, a working-day-long mean exposure to O_3_ is sufficient to cause respiratory distress [25]. Indeed, occupational health researchers codified specific building-associated illnesses, mainly consisting of respiratory disorders [6,26,27]. Pollutants (in particular PM) have also been related to neurotoxicity/dementia, atherosclerosis, gut microbiome alterations, infertility, and diabetes mellitus [11,28]. PM_2.5_ is thought to be heavily connected to sudden arrhythmic deaths, since even an acute elevated exposure can increase the risk of atrial fibrillation [5]. During the SARS-CoV-2 pandemic, several authors postulated an association between the elevated mortality rate of this respiratory viral infection and the higher levels of PM_10_ concentrations recorded in specific Italian regions relative to other areas [29]. However, from a medico-legal perspective, the possible relationship with cancer is of high importance and has been reported for many pollutants, such as micro- and nanoplastics, PM and fibers [11,25]. If some tendential correlations are known (for instance, between radon and lung carcinoma—especially in smokers—and between formaldehyde and leukemia/nasopharyngeal cancer), a very strong (but not exclusive) relationship exists between malignant mesothelioma and asbestos [21,30]. Finally, some pollutants are poisons in a strict sense, as in the case of CO, which is produced through incomplete combustion (e.g., infiltration of outdoor air from dense traffic districts, tobacco smoking, garages, poorly maintained/malfunctioning heating devices) and whose acute/long-term exposure may cause fatal asphyxia because of the inability of hemoglobin to link oxygen [1].

### 3.4. Autopsy and Histopathological Findings Potentially Linked to Indoor Air Pollution Exposure

Indoor environments account for a substantial share of lifetime exposure to pollutants because people spend most of their time in homes, workplaces, schools, and vehicles. Indoor air quality reflects the infiltration of both outdoor and indoor sources of pollution (solid-fuel combustion, tobacco smoke, office/consumer products, volatile organic compounds, and nitrogen oxides), producing complex mixtures of gases and particulate matter. Fine and ultrafine particles can reach the alveoli, interact with epithelial–endothelial barriers, and contribute to systemic oxidative and inflammatory tissue injury [31]. Although indoor pollution typically results in chronic health issues from long-term exposure, certain substances can cause immediate lethality.

#### 3.4.1. Effects on the Nervous System

Neuropathological effects have been investigated mainly in chronically exposed urban settings relevant to indoor exposure via infiltration and shared combustion sources. In exposed children and young adults, gross brain examination may be unremarkable, but targeted lesions can be detected with directed sampling of the olfactory pathways and microvasculature. At autopsy, olfactory bulbs (OB) show a distinctive black particulate material within the cytoplasm of neurons around glomeruli, consistent with particulate deposition despite the absence of gross abnormalities [32].

Ultrastructurally, OB microvessels can show endothelial hyperplasia, endothelial vacuolization, inflammatory cells adherent to the endothelium, and enlarged Virchow–Robin spaces, supporting a perivascular injury pattern [33].

In chronically exposed children/young adults, neuropathology includes disruption of the blood–brain barrier (BBB) with tight-junction abnormalities (e.g., ZO-1 alterations) and extravascular prothrombin immunoreactivity, indicating leakage of plasma proteins into the parenchyma/perivascular space. In addition, ultrafine particulate deposition in association with cerebral microvessels and inflammatory/innate immune activation (e.g., perivascular macrophage markers) has been described, linking particulate exposure to endothelial and perivascular responses. In parallel animal evidence, young dogs exposed to polluted environments show BBB alterations, reactive astroglial responses, and apoptosis of white-matter glial cells, with vascular-associated changes such as ApoE immunoreactivity in vascular smooth muscle cells and pericytes [32,33].

Post-mortem human brain tissue from severely exposed individuals shows inflammatory pathway activation (e.g., COX-2-related changes) and Aβ42 immunoreactivity involving neurons/astrocytes and vascular elements in affected regions (including cortex/hippocampal areas). In exposed children and young adults, OB pathology includes Aβ42 immunoreactivity in olfactory ensheathing cells, astrocytes, neurons, and vascular cells (arteriolar smooth muscle and capillary endothelium); isolated diffuse Aβ42 plaques can be observed even in teenagers/young adults. α-synuclein abnormalities have also been reported in OB (granular deposits across OB layers and Lewy neurites in a subset), whereas PHF-tau staining may be absent. Canine data further report non-neuritic plaques and neurofibrillary tangles alongside neuroinflammation, reinforcing a convergent proteinopathy signal across species in chronic exposure models [32,33].

In a chronic rat exposure model, air pollution exposure is associated with measurable oxidative stress (e.g., higher malondialdehyde and reduced superoxide dismutase activity in the cortex across exposed groups, with region-specific antioxidant patterns) and stereology-based differences in neuronal and microglial cell numbers (notably in the cortex depending on exposure window) [34]. Reviews integrating autopsy and experimental literature emphasize that particulate exposures can drive microglial activation, oxidative injury, and BBB-related vulnerability, including diesel exhaust particulate-induced microglia-dependent neurotoxicity (ROS/NADPH oxidase pathways) and autopsy signals such as increased CD14 expression in chronically exposed humans [34,35]. To summarize, inhaled particulates that reach the olfactory pathways and cerebral microvasculature can disrupt the blood–brain barrier, trigger microglial activation, and promote protein misfolding processes involving amyloid-β and α-synuclein, thereby fostering neuroinflammation and neurodegenerative change. These processes may manifest clinically as cognitive decline or dementia and could contribute to autonomic imbalance and arrhythmogenic susceptibility in a subset of exposed individuals, raising the theoretical possibility that chronic air-pollution-mediated brain injury indirectly influences the risk of sudden unexpected death [36,37]. From a forensic perspective, however, the neuropathological findings described are highly non-specific and overlap extensively with changes related to aging, vascular risk factors, primary neurodegenerative diseases, and other environmental exposures, which severely limits their standalone value for attributing individual deaths to indoor particulate exposure.

#### 3.4.2. Effects on the Cardiovascular System

Autopsy-relevant cardiovascular tissue evidence linking particulate air pollution to structural myocardial injury derives mainly from controlled animal models rather than large human forensic autopsy series. Specifically, absolute heart weight increases, with a statistically significant change also in the heart/body-weight ratio. Histologically, the dominant lesion is myocardial fibrosis: Picrosirius red staining shows a 166% increase in cardiac collagen deposition in PM_2.5_-exposed mice, with concomitant upregulation of profibrotic markers (increased TGF-β and collagen I) and increased collagen I protein [38]. From a mechanistic standpoint, fine particulate exposure promotes myocardial fibrosis and adverse remodeling via sustained oxidative stress and TGF-β–driven profibrotic signaling [39,40]. These structural changes plausibly lower the threshold for lethal arrhythmias and heart failure decompensation in susceptible individuals, especially in the setting of coexisting coronary artery disease or hypertension. From a forensic perspective, such remodeling is intrinsically non-specific and indistinguishable from fibrosis caused by other chronic insults (e.g., hypertension, ischemic heart disease, cardiomyopathies), which is why it can provide substrate-level support for certifying sudden cardiac death of natural cause.

#### 3.4.3. Effects on the Respiratory System

Inhaled particulate matter and irritant gases can induce a spectrum of epithelial injury and “adaptation” in the conducting airways, typically reported as mucous cell hyperplasia, squamous metaplasia, and submucosal inflammation. Still, these features are shared by many chronic inhalational insults [41,42]. In experimental inhalation mimicking high-emission indoor environments (e.g., printing/office-related aerosol mixtures), tracheal sections have been reported to show epithelial desquamation, basal cell hyperplasia, inflammatory infiltrates, and structural changes in airway wall components (including cartilage alterations) together with increased local inflammatory signaling [42]. Even when described as “pollution-related,” these changes remain non-specific because similar tracheobronchial remodeling is well documented in chronic bronchitis from tobacco smoke and other occupational exposures [41].

Autopsy-based studies repeatedly point to small airway remodeling as a structural substrate for exposure to high particulate matter loads (which can occur both outdoors and indoors in poorly ventilated settings) [42,43]. In never-smokers chronically exposed to high particulate matter, lung histology graded on Movat’s pentachrome has shown increased mural fibrosis and increased mural smooth muscle in both membranous and respiratory bronchioles, often accompanied by visible pigmented dust within or adjacent to bronchiolar walls. Notably, the visible black pigment in bronchiolar walls corresponds to combustion-type carbonaceous ultrafine aggregates retained in airway mucosa, supporting a mechanistic link between inhaled soot-like particles and airway wall retention [42].

In forensic autopsies of residents of high particulate matter environments, reported bronchiolar findings include bronchiolar secretory hyperplasia and increased acidic, rigid mucus, interpreted as adaptive responses to chronic inhalational injury. Pigment patterns can differ by exposure context: smokers typically show brown macrophages (“smoker’s pigment”) in respiratory bronchioles and adjacent alveolar ducts, whereas high particulate matter-exposed groups have been described as having greater numbers of pigment-laden macrophages and extracellular carbon pigment in bronchiolar walls (particularly respiratory bronchioles) [41,42]. However, the pigment deposition may reflect multiple sources (fossil fuel combustion, agricultural burning/wood smoke, cigarette smoke) [41,43].

During extreme combustion aerosol exposure, airway lumina may contain mucopurulent exudates with dense aggregates of opaque particulate matter within bronchioles on H&E, reflecting recent inhalation and impaired clearance. Detailed microanalysis of autopsy lung from the London smog era showed carbonaceous matrices containing numerous inorganic particles, including “heavy metal” types (reported with Pb, Zn, Sn, Fe, Sb, and occasionally Mn, Cu, Cd), demonstrating that what is seen histologically as “dust” can represent a complex indoor and outdoor mixture. The same approach emphasized compartmental patterns (airway aggregates vs. macrophage/interstitial/lymph node compartments) consistent with differing residence times and clearance pathways, a useful conceptual model when interpreting autopsy particle burdens [44].

Pleural anthracosis has been proposed as an autopsy-level indicator of cumulative particle exposure, potentially applicable to long-term indoor smoke exposure when the indoor source dominates lifetime dose (e.g., household biomass). Yet pleural pigment is also common with urban traffic pollution and occupational dust, so it is best viewed as a non-specific integrative marker rather than evidence of a particular indoor source [43].

Experimental models broaden the indoor relevance: rats exposed for 30 days to poorly ventilated, high-printing indoor air showed lung focal inflammatory infiltrates, vascular plethora, vascular sclerosis, and focal emphysema, alongside altered cytokine expression and increased apoptosis signals. Separately, the sub-chronic exposure of rats to real-world urban PM_2.5_ was associated with reduced alveolar lumen, increased alveolar macrophages, and increased PAS-positive cells, consistent with pollutant-triggered airway and alveolar injury [45].

From a mechanistic standpoint, chronic exposure to fine and ultrafine particulate matter contributes to small-airway remodeling through persistent epithelial injury, mucous cell hyperplasia, and wall fibrosis, with concurrent pigment deposition reflecting impaired clearance of inhaled soot-like particles. These changes promote airflow limitation, mucus plugging, and increased susceptibility to infectious and non-infectious exacerbations of chronic obstructive and asthmatic disease, thereby lowering the threshold for episodes of acute respiratory failure in vulnerable individuals [46]. In forensic terms, such bronchiolar and parenchymal alterations can support the certification of a natural manner of death in cases of COPD or asthma exacerbation temporally associated with periods of elevated indoor particulate burden, typically with indoor pollution listed as a contributing condition rather than as the immediate cause; however, they remain intrinsically non-specific and largely indistinguishable from lesions driven by tobacco smoke, occupational dusts, or other chronic inhalational insults.

#### 3.4.4. Effects on the Kidney and Liver

Although direct forensic autopsy case series specifically attributing renal or hepatic lesions to indoor air pollution are still limited, the available tissue-based literature supports plausible target-organ patterns that can be documented histologically in medico-legal practice. An extensive native renal biopsy registry from China reported a marked temporal increase in membranous nephropathy (MN) and an exposure–response association with long-term PM_2.5_, suggesting that chronic particulate exposure may contribute to immune-mediated glomerular injury [47]. In parallel, toxicopathology evidence indicates that inhalable pollutant constituents—particularly combustion-associated metals—can generate recognizable renal micro-lesions: arsenic-related tubulointerstitial nephritis/acute tubular necrosis (with possible nephrocalcinosis or papillary necrosis), cadmium-driven proximal tubular dysfunction, and mercury-predominant proximal tubular ultrastructural injury (loss of microvilli, organelle damage, epithelial detachment) [48].

For the liver, the dominant histopathologic endpoints linked to inhaled pollutants cluster within the NAFLD/NASH spectrum (steatosis with inflammatory and fibrotic progression), with experimental evidence that second-hand smoke can promote steatogenesis and that particulate exposures can amplify hepatic inflammation and fibrosis through Kupffer-cell–mediated pathways [49].

These organ-specific alterations typically evolve over years and rarely act as immediate causes of death; rather, they modulate the baseline risk of fatal events by predisposing to progressive renal failure, increased vulnerability to sepsis, and a higher burden of cardiometabolic disease. In forensic practice, such chronic renal and hepatic lesions can therefore be interpreted as part of a broader comorbidity profile that may have amplified the impact of an acute stressor or intercurrent illness, but their non-specific nature and the frequent coexistence of stronger causal factors (e.g., diabetes, hypertension, alcohol use, autoimmune disease) make it difficult to ascribe an individual death directly to indoor air pollution on the basis of these findings alone.

#### 3.4.5. Effects on Pregnancy

Pregnancy is a particularly vulnerable window for indoor air pollution exposures because maternal inhalation can translate into placental dysfunction and impaired fetal growth. Systematic reviews and meta-analyses link indoor pollution exposure with higher risks of low birth weight, preterm birth, stillbirth, and fetal growth restriction [50,51].

Beyond epidemiologic outcomes, placental tissue studies provide mechanistic anchors: in a Tanzanian cohort with personal monitoring of PM_2.5_ and CO during pregnancy and placental examination after delivery, higher household air pollution exposure was associated with specific placental pathology patterns, and fetal thrombosis might contribute to adverse outcomes [52]. Importantly, human evidence also supports direct particle materno–fetal interface interaction: black carbon particles have been detected on the fetal side of human placentas, suggesting translocation of inhaled carbonaceous particles toward the fetus [53]. Extending this concept, subsequent work reported the presence of carbonaceous particles in human fetal organs during gestation, thereby reinforcing the biological plausibility for early-life tissue exposure [24].

From a medico-legal standpoint, however, death certificates in such cases typically record fetal asphyxia, complications of prematurity, or unexplained intrauterine fetal demise as the underlying cause of death, with indoor smoke or particulate exposure, when documented, best framed as a contributing factor within a multifactorial risk landscape that also includes maternal disease, nutritional status, and other environmental and social determinants.

#### 3.4.6. Acute Lethal Effects

Although most indoor pollution effects manifest chronically through cumulative exposure, certain agents such as CO and hydrogen cyanide (HCN) can trigger rapid lethality. CO, resulting from incomplete combustion (e.g., faulty heaters, stoves, enclosed garages), binds to hemoglobin with an affinity 200 times greater than oxygen, inducing severe tissue hypoxia. Post-mortem diagnosis is supported by femoral blood carboxyhemoglobin (COHb) levels > 50% (compared to <10% physiological levels), with cherry-red livor mortis and cerebral edema serving as diagnostic hallmarks [22]. Similarly, HCN—released during the combustion of plastics, polyurethanes, or electroplating chemicals (e.g., cyanide salts reacting with acids in confined spaces)—inhibits cytochrome oxidase, leading to histotoxic hypoxia. Post-mortem blood HCN concentrations exceeding 3 μg/mL are typically lethal (toxic levels > 1 μg/mL). Key forensic findings include pink livor mortis, petechial hemorrhages (heart/lungs), pulmonary edema, and a bitter almond odor, though the latter is not consistently detectable [54]. Differential diagnosis includes cardiovascular events and other intoxications.

Non-chemical acute outcomes include IgE-mediated anaphylactic shock from molds or allergens (indicated by post-mortem tryptase > 20 ng/mL and laryngeal edema) or severe irritant responses to VOCs/ammonia (chemical pneumonitis, ARDS-like) [55,56,57]. Investigation necessitates rigorous scene sampling, toxicology, and clinical history. These rare events require immediate post-mortem biomarkers to differentiate environmental fatalities from natural causes or iatrogenic factors, ultimately supporting the certification of the manner of death as accidental versus homicidal.

#### 3.4.7. Vulnerable Populations and Gender Differences

Vulnerable groups, such as children, elderly individuals, and low-income populations, experience a disproportionate disease burden driven by age, comorbidities, and social determinants, complicating isolation of indoor air pollution’s contribution in forensic casework [10,58]. Pediatric cases often show acute respiratory distress and small-airway disease, amplified by higher minute ventilation per kilogram of body weight and ongoing lung development, but overlapping with prevalent endogenous morbidity [59]. Elderly individuals exhibit chronic obstructive pulmonary disease exacerbations and cardiac decompensation superimposed on coronary artery disease or heart failure, indistinguishable from aging and cardiorespiratory natural history [60]. Low-income settings are characterized by substandard housing, poorly ventilated workplaces, and malnutrition, concentrating competing risks such as smoking and infections, where indoor pollution acts as a contributory rather than a proximate cause [61]. Gender-specific risks also emerge, particularly in developing countries with marked traditional roles [62]. Women face higher domestic risks from biomass fuel smoke in poorly ventilated kitchens, while men are more frequently exposed to occupational hazards, such as welding fumes in industrial workshops [63]. However, stratifying by allergic disease resulted in no significant sex difference in symptom reporting from indoor air pollution [64]. Findings from cross-sectional studies, such as those in Sarnia’s “Chemical Valley,” suggest inverse age–exposure correlations, urging caution when interpreting sex-symptom links, as occupational co-exposures and pre-existing conditions significantly modify outcomes [64].

These data are summarized in Table 1.

### 3.5. Dose–Response Relationships Research

In clinical research, dose–response assessment aims to evaluate the risk of those who are exposed to a pollutant. The risk is assessed by taking into account the qualitative and quantitative relationship between exposure and health outcome and characterizing the exposure in terms of duration, frequency, and magnitude [65]. In general terms of clinical/occupational toxicology, the dose–response assessment process consists of selecting a study, exposure metrics, a benchmark response (a biologically relevant/measurable response to a specific dose), and a suitable mathematical model, then identifying the point of departure and finally calculating toxicity values [65].

Over the last three decades, dose–response studies on air pollutants have mainly focused on outdoor pollution and on specific chemicals such as PM_2.5_, O_3_, and NO_2_ [66,67,68,69,70,71,72]. Chronic exposure to PM_2.5_ was reported to increase mortality, with a significant monotonic dose–response relationship in almost all the deciles [66]. A positive dose–mortality relationship was also found for chronic exposure to NO_2_ (but for exposure to low concentrations) and O_3_ [66].

That being said, a causal relationship relies on two pillars: (i) factual evidence—i.e., proof that, in practice, A actually caused B; and (ii) counterfactual evidence—i.e., proof that, in theory, if A did not happen, B would not have occurred. In observational studies, using a counterfactual framework is extremely challenging. If exposure is considered a binary variable (exposed vs. unexposed), a statistical approximation may be achieved to ponderate the exposure effect, artificially creating a balanced pseudo-population by using the weight of the inverse of the probability of the observed exposure (i.e., “inverse probability weighting” method) [73]. When exposure is considered a continuous variable, Wei et al. also proposed to bin the study population into deciles (ten same-size groups), with the decile corresponding to the lowest exposure used as a reference (i.e., “decile binning approach”) [66].

## 4. Discussion

Indoor air pollution can be partly prevented through interventions—such as regular ventilation, air cleaners, smoking bans enforcement, phytoremediation, and avoiding immediately occupying recently constructed/renovated buildings [1,2,25,74]. Some of these interventions, such as ventilation, are regulated at the national, supranational (e.g., European Union), and/or international (e.g., World Health Organization) levels, as the upper limits of many pollutants [7]. However, international and European Union laws tend to focus on outdoor air pollution and are highly fragmented and poorly implemented [7,75]. Given these regulatory limitations, a core issue is to sanction transgressors, since enforcing regulations and sanctioning transgressors are two sides of the same coin. In Western countries, criminal liability is personal (i.e., only the person actually is responsible for the wrongful conduct can be considered liable, and liability cannot be shared/fractionated) and depends on proving misconduct and its consequences beyond any reasonable doubt. Instead, civil liability can be shared/fractionated and requires that a specific causal explanation prevails over others in terms of likelihood.

However, relating an effect (such as death) to a cause (such as exposure to a specific pollutant) is extremely complex: conducting prospective case–control studies would be unethical and unfeasible, and the current evidence is largely limited to observational research that is associational (rather than causal) [66,76]. Indeed, the scope of epidemiological research differs in nature from that of a forensic investigation: dose–response relationships are used to determine if a target level of pollutants may significantly impact health rather than to establish if human conduct could impact the chances of developing a disease [66]. Moreover, as shown in our paper, research models can be biased in both the factual and the counterfactual frameworks [66,77]. Besides statistical limitations (i.e., there is no experimental evidence), there are many possible biases when scientific evidence is translated into court. First, most of the studies that evaluated the mortality caused by pollution focused on outdoor pollution, but each indoor environment is unique and generally very different in composition and levels of pollutants from the outdoor environment [1,2,11]. Possible arguments advocating for using epidemiological research are that indoor environments are often more polluted than outdoor environments and that epidemiological biases in some cases lead to underestimating (rather than overestimating) the actual strength of the association between exposure and biological response [1,2,11,77]. However, other issues should be taken into account and discussed in a medico-legal analysis. For instance, the possible interference/uncertainty arising from the interplay between multiple pollutants and the effects of endocrine-disrupting chemicals (such as lead or brominated flame retardants) that tend to show abnormal dose–response relationships with a non-monotonic pattern or even effects below the lowest observed adverse effect level [78]. In detail, endocrine-disrupting chemicals are chemicals that can mimic, inhibit, or interfere with natural hormones. It is likely that their often-abnormal dose–response relationships are due to their chemical similarity to hormones, which can show non-monotonic dose response depending on different variables such as the tested tissue [79]. Possible explanations of non-monotonic responses include the fact that hormone signaling is cell-, tissue- and organ-specific; the dose-dependent affinity to different receptors; and the dose-dependent response to the activation of a specific receptor [79,80]. In general terms, the assumption behind the concept of “safe dose” is that low doses of a chemical may have predictable, negligible effects, although for some chemicals (e.g., mutagens and carcinogens) it is commonly accepted that there is no safety threshold [81]. Indeed, the risk assessment frameworks usually derive from evidence obtained in contexts of exposure to high doses and postulate a linear dose–response relationship [82]. However, below the threshold, there can still be biological responses: some substances at low doses may still cause negative effects (e.g., stimulate microbial activity) or even produce positive responses (the so-called “hormesis”) [82]. Moreover, humans are often exposed to mixtures of chemicals rather than to single substances, and multiple pollutants may cause additive, positive synergistic, negative synergistic, positive antagonistic, or negative antagonistic effects [83]. The translation of general assumptions into real-world case analysis is then highly biased, because in many cases regulations only focus on individual chemicals, the mechanisms of action are unknown, the possible interactions (in terms of magnitude and nature) between chemicals—even at very low individual doses—are unknown (and cannot be postulated), there are no relevant data from human studies, and overall, it is unclear whether the toxicity depends on the total mixture dose or its component proportions [84]. In response to these limitations, a common regulatory approach is to postulate a no-interaction model that is often unrealistic and thus of no interest for forensic analysis [83].

Additionally, many other variables may interfere: for instance, temperature and relative humidity can promote the release of organic pollutants from materials, making it very difficult to infer actual exposure over a given time [1]. Even genes can play an important role; for instance, regarding mesothelioma, mutations that impede mesothelial cell death or that make cells particularly sensitive to fibers may significantly favor cancerogenesis [12]. In practice, if in civil proceedings these limitations could be overridden by a careful and meticulous combined analysis of scientific literature and data specifically obtained from the discussed case, while in criminal proceedings it could be arduous to prove guilt beyond any reasonable doubt. Indeed, in a case of lung cancer in an employee who worked in a polluted workplace, it could be difficult to prove that it was caused by the contaminating asbestos rather than—among other causes—by external environment asbestos or by one of the more than 400 unregulated minerals (e.g., erionite, which is carcinogenic) with comparable physical and chemical properties that are present in the natural environment [12].

Furthermore, many dose–response relationships are far from being linear/homogeneous, which makes it very difficult to establish who among multiple employers is criminally liable for a pollutant-related disease manifested by a worker at the end of his/her career. For instance, the risk of mesothelioma is highest for those who, in the first years of their career, were exposed to high doses of asbestos and, for the rest of their job history, were exposed to low levels [85]. Moreover, Forastiere et al. reported that exposure to low-to-moderate concentrations of PM_10_ was the strongest death predictor [86]. Finally, causation in pollution is based on the paradigm of linear dose–response relationships: above a certain threshold (the so-called “no-observed-adverse-effect-level”), the higher the dose, the stronger the expected response, while below this threshold, there should be no biological response, or, at least, no statistically resolvable response [82]. First, it is debatable what biological response should be considered: subcellular, cellular, tissue or organic [87,88]. However, we do know that non-linear dose–response models better approximate reality because there can be (even beneficial) responses below the threshold [82,89]. For instance, some substances (such as heavy metals and micro- or nanoparticles) may induce biphasic dose-responses (i.e., hormetic responses) [90]. Hence, causal inference cannot be based on linear cause–effect models, and new models (interpreted with the help of occupational health experts/specialized toxicologists) should be adopted.

Given all these limitations, it is clear that a prominent role should be played by autopsy, which, by definition, is the forensic investigation aimed at gathering specific information. However, as shown by our paper, especially in sudden deaths, it is extremely likely that the autopsy will fail to identify specific or definitive anomalies useful to bridge the gap between general knowledge from the literature and the real-world case. Indeed, the abnormalities observed at autopsy are often highly non-specific and may result from various alternative pollutants or even non-toxic factors (e.g., fibrosis can derive from chronic inflammatory states due to pathogenic genotypes or infectious diseases). Across organs, pollution-related tissue responses largely converge on shared injury pathways, such as oxidative stress, endothelial dysfunction, and inflammation, rather than producing pathognomonic lesions. Particulate matter may translocate into the bloodstream and reach multiple extrapulmonary tissues, leading to findings such as airway pigment deposition and bronchiolitis, myocardial fibrosis and remodeling, neuroinflammation, glomerular immune-complex deposition, or steatohepatitis-like changes. These alterations are intrinsically non-specific, overlapping with those caused by smoking, infection, metabolic syndrome, ischemia, drugs, and numerous other conditions. Therefore, in medico-legal practice, any attribution to indoor air pollution requires a thorough reconstruction of the environmental context and exposure, careful exclusion of alternative etiologies, and correlation of histological findings with clinical background and comorbidities.

**Figure 1:**
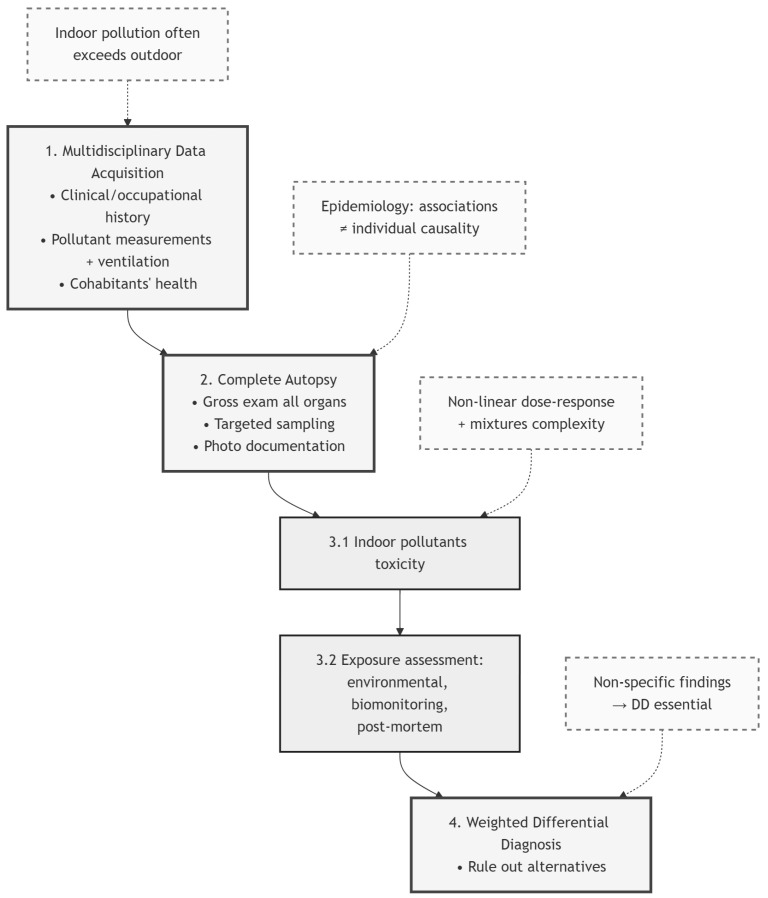
Stepwise multidisciplinary forensic workflow for investigating indoor air pollution-related fatalities.

In conclusion, we propose the following step-by-step approach for forensic investigations aimed at determining whether indoor pollutants contributed to death (as graphically summarized in Figure 1):

- Multidisciplinary Data Acquisition: Collect all available information regarding the deceased’s medical history and the scene of death. This must include detailed documentation and photographs of the building type, indoor air quality data (from the primary and adjacent rooms), and ventilation parameters. Furthermore, the health status of cohabitants or colleagues should be assessed for shared symptoms.
- Case Discussion and Matrix Selection: Evaluate the preliminary findings within a multidisciplinary team, including a forensic pathologist and a toxicologist. This collaboration is essential to define the analytical strategy and select the appropriate matrices during the autopsy. At a minimum, peripheral and central blood, urine, hair, liver, and kidney samples should be collected.
- Comprehensive Autopsy and Targeted Sampling: Perform a complete post-mortem examination, with specific focus on the brain, heart, respiratory system, liver, and kidneys to identify macro- and microscopic markers of toxicity.
- Weighted Differential Diagnosis: If the autopsy reveals signs compatible with potentially lethal indoor intoxication, all plausible explanatory hypotheses must be scientifically weighed. These findings should be accompanied by a multidisciplinary commentary to assist the judicial authority in determining whether a single causal pathway is reasonable or if one hypothesis prevails on a “balance of probabilities” (probabilistic prevalence).

## 5. Conclusions

Indoor air pollution requires deep medico-legal evaluation, since prospective studies are unfeasible for ethical reasons and current evidence, used for epidemiological purposes, remains largely associational. Relating death or disease to specific indoor exposures is extremely complex, with biases in factual/counterfactual frameworks, unique indoor pollutant profiles, multi-pollutant interactions, and non-linear dose responses complicating causal inference.

Moreover, considering potential confounding factors from genetics or environment, establishing criminal liability for indoor air pollution remains arduous in Western countries where proving guilt is personal and depends on proving misconduct and its consequences beyond any reasonable doubt. While autopsy plays a prominent role, this review highlights the opportunity to involve occupational health experts and specialized toxicologists to explore new causal inference models addressing these evidentiary gaps.

## Figures and Tables

**Table 1:** Pathological findings, mechanisms, and forensic implications of indoor air pollution exposure.

	Key Pathological Alterations	Main Pathophysiological Mechanisms	Plausible Forensic Cause of Death
Central nervous system	Particulate deposition in olfactory bulbs; microvascular endothelial hyperplasia/vacuolization; enlarged Virchow-Robin spaces; BBB disruption with ZO-1 alterations; Aβ42/α-synuclein immunoreactivity; oxidative stress markers	BBB permeability ↑, microglial activation, neuroinflammation, protein misfolding (Aβ42, α-synuclein), oxidative stress (MDA ↑, SOD ↓)	Dementia/cognitive decline (natural); possible SUDEP/autonomic death (natural)
Cardiovascular system	Increased heart/body-weight ratio; interstitial myocardial fibrosis (Picrosirius red + collagen ↑); profibrotic markers (TGF-β, collagen I)	Oxidative stress, TGF-β/Smad profibrotic signaling, adverse remodeling	Sudden cardiac death (natural); heart failure decompensation (natural)
Respiratory system	Small-airway remodeling (fibrosis, smooth muscle ↑); mucous hyperplasia; pigment-laden macrophages; pleural anthracosis; mucopurulent exudates (acute)	Epithelial injury, impaired mucociliary clearance, chronic inflammation/fibrosis, gas-exchange impairment	COPD/asthma exacerbation; acute respiratory failure (natural); inhalation injury (accidental)
Kidney	Membranous nephropathy; tubulointerstitial nephritis; proximal tubular injury (metals)	Immune-complex deposition, metal-induced tubular toxicity, oxidative damage	Chronic renal failure, sepsis predisposition (natural)
Liver	Steatosis, inflammation, fibrosis (NAFLD/NASH-like)	Steatogenesis, Kupffer-cell mediated inflammation/fibrosis	Hepatic failure, cardiometabolic decompensation (natural)
Pregnancy	Placental vascular changes; fetal thrombosis; black carbon in fetal tissues	Placental hypoxia, thrombosis, particle translocation	Stillbirth, preterm delivery complications, IUFD (natural)

↑ increased; ↓ decreased.

## Data Availability

The data supporting the findings of this study are available from the corresponding author upon reasonable request.
